# Construction and characterization of a hypervesiculation strain of *Escherichia coli* Nissle 1917

**DOI:** 10.1371/journal.pone.0301613

**Published:** 2024-04-02

**Authors:** Tomomi Sawabe, Yoshihiro Ojima, Mao Nakagawa, Toru Sawada, Yuhei O. Tahara, Makoto Miyata, Masayuki Azuma

**Affiliations:** 1 Department of Chemistry and Bioengineering, Graduate School of Engineering, Osaka Metropolitan University, Osaka, Japan; 2 Graduate School of Science, Osaka Metropolitan University, Osaka, Japan; Kohat University of Science and Technology, PAKISTAN

## Abstract

Outer membrane vesicles (OMVs) are produced by Gram-negative bacteria and deliver microbial molecules to distant target cells in a host. OMVs secreted by probiotic probiotic strain *Escherichia coli* Nissle 1917 (EcN) have been reported to induce an immune response. In this study, we aimed to increase the OMV production of EcN. The double gene knockout of *mlaE* and *nlpI* was conducted in EcN because the Δ*mlaE*Δ*nlpI* of experimental strain *E*. *coli* K12 showed the highest OMV production in our previous report. The Δ*mlaE*Δ*nlpI* of EcN showed approximately 8 times higher OMV production compared with the parental (wild-type) strain. Quick-freeze, deep-etch replica electron microscopy revealed that plasmolysis occurred in the elongated Δ*mlaE*Δ*nlpI* cells and the peptidoglycan (PG) had numerous holes. While these phenomena are similar to the findings for the Δ*mlaE*Δ*nlpI* of K12, there were more PG holes in the Δ*mlaE*Δ*nlpI* of EcN than the K12 strain, which were observed not only at the tip of the long axis but also in the whole PG structure. Further analysis clarified that the viability of Δ*mlaE*Δ*nlpI* of EcN decreased compared with that of the wild-type. Although the amount of PG in Δ*mlaE*Δ*nlpI* cells was about half of that in wild-type, the components of amino acids in PG did not change in Δ*mlaE*Δ*nlpI*. Although the viability decreased compared to the wild-type, the Δ*mlaE*Δ*nlpI* grew in normal culture conditions. The hypervesiculation strain constructed here is expected to be used as an enhanced probiotic strain.

## Introduction

Extracellular vesicles isolated from Gram-negative bacteria, called outer membrane vesicles (OMVs), have nanosized, spherical, bilayered membranous structures containing outer membrane proteins, lipids, periplasmic components, lipopolysaccharides, RNA, and DNA [1, 2]. OMVs have various described roles, including as decoys for viral and antibiotic attacks, in quorum sensing, and in regulation of host immune defense [3].

*Escherichia coli* Nissle 1917 (EcN) is one of the most common Gram-negative probiotic strains [4] and is marketed in several countries as the drug product “Mutaflor” (Ardeypharm, Herdecke, Germany) [5, 6]; it is widely used to treat enteric diseases, including infectious diarrhea and inflammatory bowel disease. In addition, EcN cells and their OMVs enhance host cell-mediated immune response, and modulate the balance between pro- and anti-inflammatory cytokines [5, 7]. Therefore, EcN OMVs are considered to be secretory products that are associated with the health effects of EcN, i.e., they are “postbiotics”. EcN OMVs are expected to be applied in various therapeutic approaches, such as vaccines, drug carriers, and regenerative medicine [8, 9].

EcN OMVs are useful, but the amount of OMVs obtained from culture is small. Several studies have shown ways to increase OMV production in experimental *E*. *coli* strains [10, 11]. Vesicle formation is promoted by external factors, e.g., growth inhibition and exposure to antibiotics, and by internal factors, e.g., metabolic turnover of cell wall components, envelope stress on the OM, and accumulation of phospholipids. In the case of EcN, OMV production was promoted by the addition of external factors such as glycine [12]. Glycine enhanced the OMV production by inhibiting peptidoglycan (PG) synthesis via substitution of glycine for DL-alanine in PG during growth of EcN, suggesting that weakening PG is important for increasing OMV production [13]. In an experimental *E*. *coli* K12 strain, our previous studies demonstrated that double gene knockout of *mlaE* and *nlpI*, which changed both the envelope structure and phospholipid accumulation properties, synergistically increased OMV production [14]. Furthermore, quick-freeze, deep-etch replica electron microscopy (QFDE-EM) revealed that PG isolated from the Δ*mlaE*Δ*nlpI* strain contained many holes, indicating that PG was damaged in this hypervesiculation strain constructed by genetic modification [15]. In addition to our report, other attempts to increase OMVs in an experimental *E*. *coli* K12 strain have been reported, such as the gene deletion of *degP*, *lpp* etc. [16]. In the case of EcN, the promoting effect of single deletion of *nlpI* on OMV production has already been reported [17]. However, quantitative data on OMV production were not shown in that paper, and the details and properties of the cells and the OMVs produced remain unclear.

In this study, we aimed to increase the OMV production of EcN. The double gene knockout of *mlaE* and *nlpI* was conducted because the Δ*mlaE*Δ*nlpI* of experimental strain *E*. *coli* K12 showed the highest OMV production in our previous report [14]. We evaluated the OMV production, and analyzed the mechanism of promotion of OMV production referring to the case of our previously reported *E*. *coli* K12 strain. There is an also report that PG contained in OMVs of EcN activates immune responses in intestinal epithelial cells [18], the structural characteristics of PG in Δ*mlaE*Δ*nlpI* of EcN cells were analyzed and discussed.

## Materials and methods

### Bacterial strains and culture conditions

Table 1 lists the strains and plasmids used in this study. EcN (serotype O6:K5:H1) was obtained from Pharma-Zentrale GmbH (Germany). Single or double gene knockout mutants were constructed by P1 transduction using the P1kc phage [14]. Kanamycin resistance was used as a selection marker. The deletion was verified by PCR using loci-specific primers (S1 Table) and its sequencing.

**Table 1:** *E*. *coli* strains used in this study.

Strain	Genotype	Reference
BW25113	*rrnB*T14 *ΔlacZ*WJ16 *hsdR*514 *ΔaraBAD*AH33	Keio collection
BW25113Δ*nlpI* (JW3132)	BW25113, Δ*nlpI*::*FRT-Km-FRT*	Keio collection
BW25113Δ*mlaE* (JW3161)	BW25113, Δ*mlaE*::*FRT-Km-FRT*	Keio collection
Nissle 1917 (EcN)	DSM 6601, serotype O6:K5:H1	Pharma-Zentrale GmbH (Germany)
EcNΔ*nlpI*	EcN, Δ*nlpI*::*FRT-Km-FRT*	This study
EcNΔ*mlaE*Δ*nlpI*	EcN, Δ*mlaE*Δ*nlpI*::*FRT-Km-FRT*	This study

Cells were cultured in lysogeny broth (LB; 10 g/L Bacto^™^ Tryptone, 5 g/L yeast extract, and 10 g/L NaCl). All the test strains were precultured in LB for 18 h at 37°C and inoculated into 100 mL of fresh LB in 500-mL baffled conical flasks to an initial optical density at 660 nm (OD_660_) of 0.01. The cultures were incubated on an NR-30 rotary shaker (Taitec, Osaka, Japan) at 140 strokes/min. Cell growth was measured using OD_660_.

### Quick-freeze, deep-etch replica EM

For QFDE-EM analysis, cells after 24 h of culture were washed twice with phosphate-buffered saline (PBS; pH 7.5) and resuspended in HEPES-NaCl buffer or 15% (v/v) glycerol solution [15, 19]. Then, centrifuged cell pellets were placed onto a rabbit lung slab, mica flakes and a paper disk attached to an aluminum disk. The samples were frozen quickly using liquid helium with a CryoPress (Valiant Instruments, St. Louis, MO, USA). The rabbit lung slab and the mica flakes function as shock absorbers in quick freezing and a flat background in observation, respectively. The specimens were stored in a chamber at −180°C using a JFDV freeze-etching device (JEOL, Tokyo, Japan). After the temperature of the sample was increased to −120°C, they were freeze-fractured with a knife and freeze-etched at −104°C for 15 min. The freeze-etching step was omitted when 15% (v/v) glycerol was used as the solvent. Subsequently, the samples were coated with platinum at a thickness of 2 nm and a rotary shadowing angle of 20° and coated with carbon at a rotary shadowing angle of 80°. Next, the replicas were floated on full-strength hydrofluoric acid, rinsed in water, cleaned with commercial bleach containing sodium hypochlorite, rinsed in water, and finally placed onto 400-mesh Cu grids. The replica specimens were observed by transmission electron microscopy (TEM) using a JEM-1010 instrument (JEOL).

### Isolation and observation of OMVs

OMVs were isolated as previously described [20] with some modifications. After incubation for 24 h, 100 mL of culture was centrifuged at 10,000 × *g* for 10 min at 4°C to remove the cells. Then, the supernatant was passed through a 0.45 μm filter. Ammonium sulfate was added at the final concentration of 400 g/L followed by incubation for 1 h at room temperature. Crude OMVs were obtained via centrifugation at 11,000 × *g* for 30 min at 4°C. The crude extracts were dissolved in 500 μL of 15% (v/v) glycerol and concentrated using a CS100FNX ultracentrifuge (Hitachi Koki Co., Tokyo, Japan) at 109,000 × *g* for 1 h. The OMV pellets were resuspended in 100 μL of 15% (v/v) glycerol. The resulting OMV samples were 1000-times more concentrated than those in the original cultures because of the volume decrease from 100 mL to 100 μL. The OMV samples were placed onto a 200-mesh copper grid and negatively stained with 4% uranyl acetate for TEM observation using a JEM-2100 instrument (JEOL).

### Evaluation of OMV production using FM4-64

OMVs were quantified with lipophilic dye FM4-64 according to a previously described method with minor modifications [15]. Isolated OMVs were incubated with 5 μg/mL FM4-64 (Molecular Probes/Thermo Fisher, Waltham, MA, USA) in PBS (pH 7.5) for 20 min. Then, OMVs were measured at the excitation and emission wavelengths of 558 and 734 nm, respectively, using an INFINITE 200 PRO spectrofluorophotometer (TECAN, Switzerland). An OMV sample without staining by FM4-64, and FM4-64 only, were used as negative controls.

### Preparation and analysis of sacculi

For the preparation of sacculi, cells at 24 h in culture were collected by centrifugation (10,000 × *g*, 4°C) for 10 min and resuspended in PBS (pH 7.5). These steps were repeated twice. Then, the cells were resuspended in 10% sodium dodecyl sulfate (w/v) and incubated at 95°C for 12 h. The resultant sacculi were harvested by centrifugation at 200,000 × *g* for 40 min, and washed three times with Milli-Q water. To determine the dry weight of total sacculi, the sacculi were lyophilized and the weight percentage of sacculi to dry cells was calculated. For determination of the dry weight of total cells, a cell pellet obtained from the same amount of culture both was washed and lyophilized. To evaluate the amino acid constituents in PG, 1 mg of lyophilized sacculi was suspended in 1 mL 6 M HCl and hydrolyzed at 95°C for 16 h. The resultant solution (1 μL) was spotted onto a thin-layer chromatography (TLC) plate; 1 μL of marker solutions of 3 mg/mL D-alanine, 3 mg/mL L-alanine, 3 mg/mL D-glutamic acid, and 10 mg/mL 2,6-diaminopimeric acid were also spotted onto the TLC plate. The development solvent was a mixture of *n*-butanol:acetic acid:distilled water (4:1:2 v:v:v). The plate was dried, ninhydrin spray was applied uniformly, and the TLC plate was warmed on a hot plate for coloring.

For the observation of sacculi by QFDE-EM, the sacculi dissolved in Milli-Q water before lyophilization were directly treated using the same procedure as described above for cells.

## Results

### Profile of OMVs from the constructed hypervesiculating strain of EcN

Although previous research reported the enhancement of OMV production in a Δ*nlpI* strain of EcN compared with the wild-type (WT) [17], quantitative results were not shown in that paper. In this study, we constructed a single gene knockout mutant, Δ*nlpI*, and double gene knockout mutant, Δ*mlaE*Δ*nlpI*, of EcN and evaluated the OMV production.

**Fig 1:**
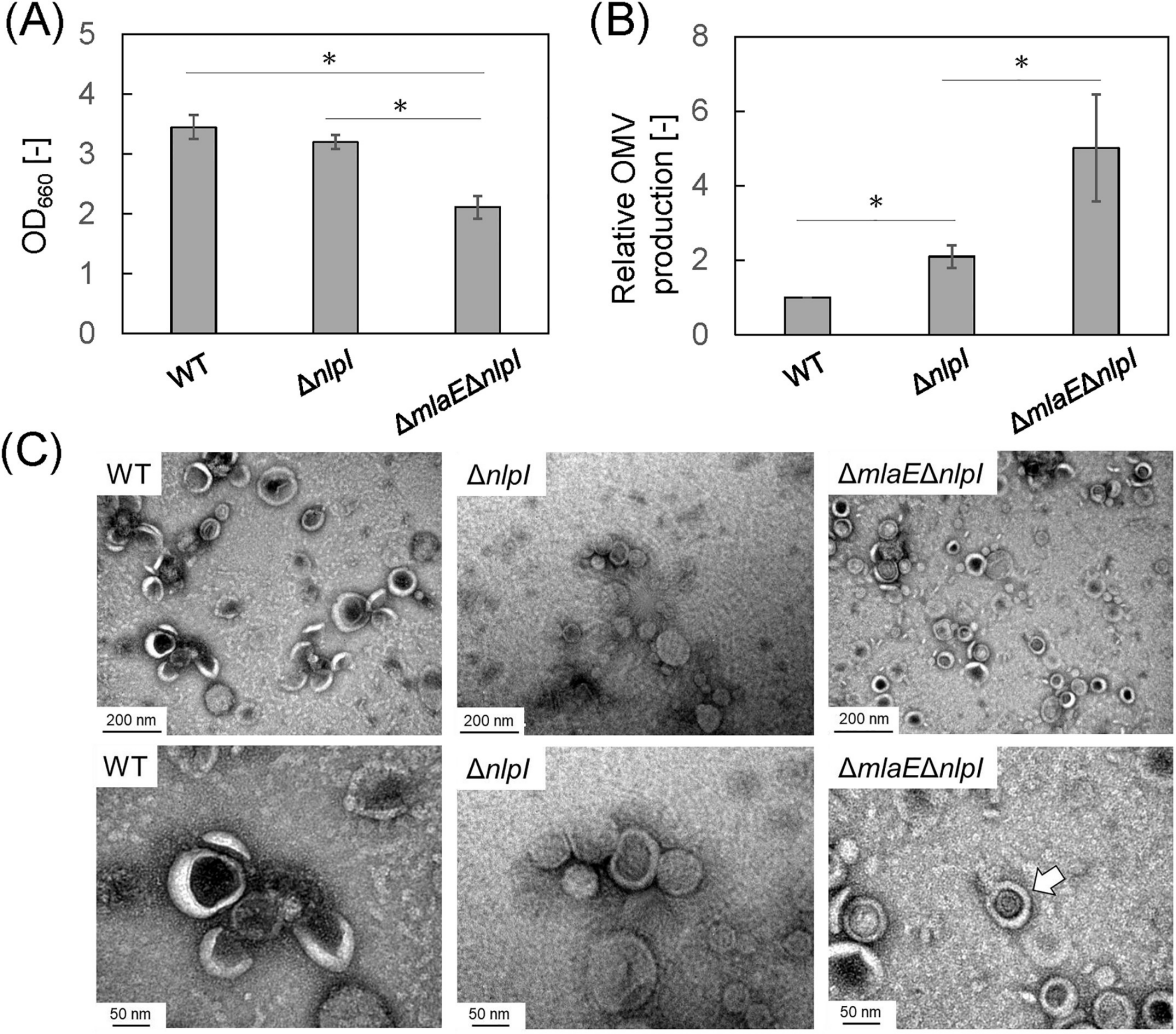
Outer membrane vesicle (OMV) production in *Escherichia coli* EcN wild-type (WT) and knockout mutants. (A) OD_660_ value of each strain (B) Relative OMV production by each strain after 24 h of culture. Vertical bars indicate standard deviations (calculated from more than three independent experiments). Statistically significant differences (*p*<0.05) are marked with asterisks. (C) Transmission electron microscopy images of OMVs isolated from the of each EcN strain. The OMVs were stained with uranyl acetate. The arrow indicates a multilamellar vesicle.

Fig 1A shows the cell growth of the single- and double gene knockout mutants of EcN after culture for 24 h. The OD_660_ of the WT strain was 3.5. The OD_660_ of the Δ*nlpI* strain was slightly decreased compared to WT. That of the double gene knockout mutant Δ*mlaE*Δ*nlpI* was 2.1 which was significantly lower than WT. These tendencies of growth suppression by gene deletion are consistent with previous results for the *E*. *coli* K12 strain BW25113 [14].

Next, OMV production by EcN was determined quantitatively by FM4-64 staining of isolated OMVs. Fig 1B shows the relative OMV production by the single and double gene knockout mutants compared with that by the EcN WT. OMV production by the Δ*nlpI* strain was 2.1-fold that by the WT (Fig 1B). The enhancement of OMV production by *nlpI* deletion was thus confirmed in EcN as in previous research [17]. However, the degree of increase was smaller than that for the Δ*nlpI* of K12 strain BW25113 (where the increase was approximately 6-fold) [14, 15]. OMV production by the Δ*mlaE*Δ*nlpI* of EcN strain was 5.0 times that by the WT. Even though the degree of increase was less than that for the K12 strain, where it was approximately 14-fold [15], enhancement of OMV production was confirmed in EcN.

Fig 1C shows negative-staining TEM images of isolated OMVs. We confirmed the OMV formation in each EcN strain. More interesting, multilamellar OMVs were observed with a low probability in the Δ*mlaE*Δ*nlpI*, but not in the case of WT or Δ*nlpI*. Multilamellar OMVs were first observed in the Δ*mlaE*Δ*nlpI* of K12 strain BW25113, and their production mechanism is closely related to plasmolysis of cells and intracellular vesicle production [15].

### Observation of WT and Δ*mlaE*Δ*nlpI* cells using QFDE-EM

**Fig 2:**
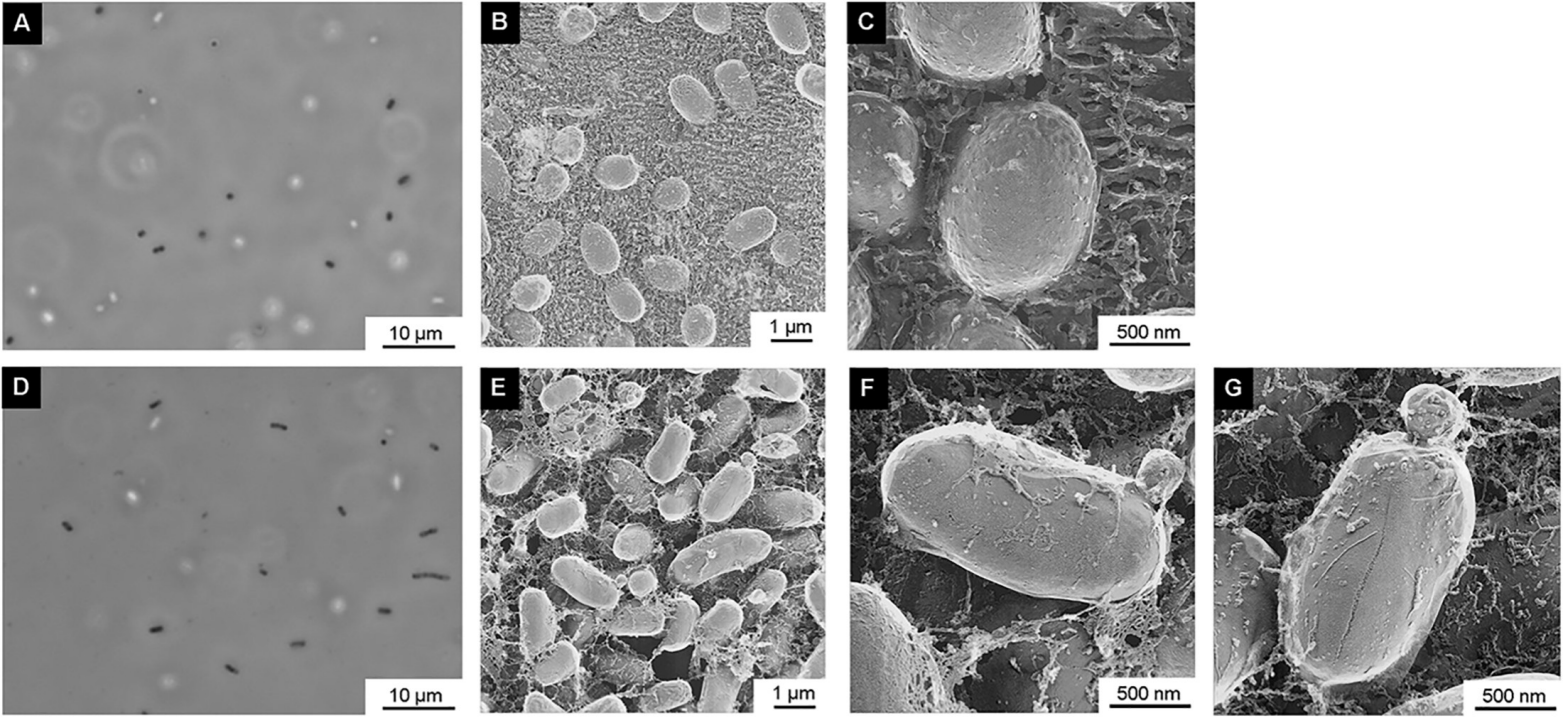
Surface structure of WT and Δ*mlaE*Δ*nlpI* of EcN cells visualized via quick-freeze, deep-etch electron microscopy (QFDE-EM). (A, D) Phase-contrast optical microscopy images of the WT (A) and Δ*mlaE*Δ*nlpI* cells (D). (B) Field image of WT cells. (C) Magnified image of the surface of WT cells. (E) Field image of Δ*mlaE*Δ*nlpI* cells. (F–G) Magnified images of the surface of Δ*mlaE*Δ*nlpI* cells.

To confirm whether plasmolysis or intracellular vesicle production occurred in Δ*mlaE*Δ*nlpI* cells of EcN, the cell structure of each strain was visualized using QFDE-EM. The shape and dimensions of the cells were consistent with the images of living cells obtained by optical microscopy for the WT (Fig 2A) and Δ*mlaE*Δ*nlpI* (Fig 2D). The surface structures of the cells were visualized by using QFDE-EM for the WT (Fig 2B and 2C) and Δ*mlaE*Δ*nlpI* (Fig 2E–2G). The Δ*mlaE*Δ*nlpI* cells seemed to be longer than WT cells (Fig 2D and 2E), and several cells were observed to form vesicles. Magnified images showed that the Δ*mlaE*Δ*nlpI* cells formed OMVs from the tip of their long axis in the same manner as K12 strain BW25113 (Fig 2F and 2G) [15].

**Fig 3:**
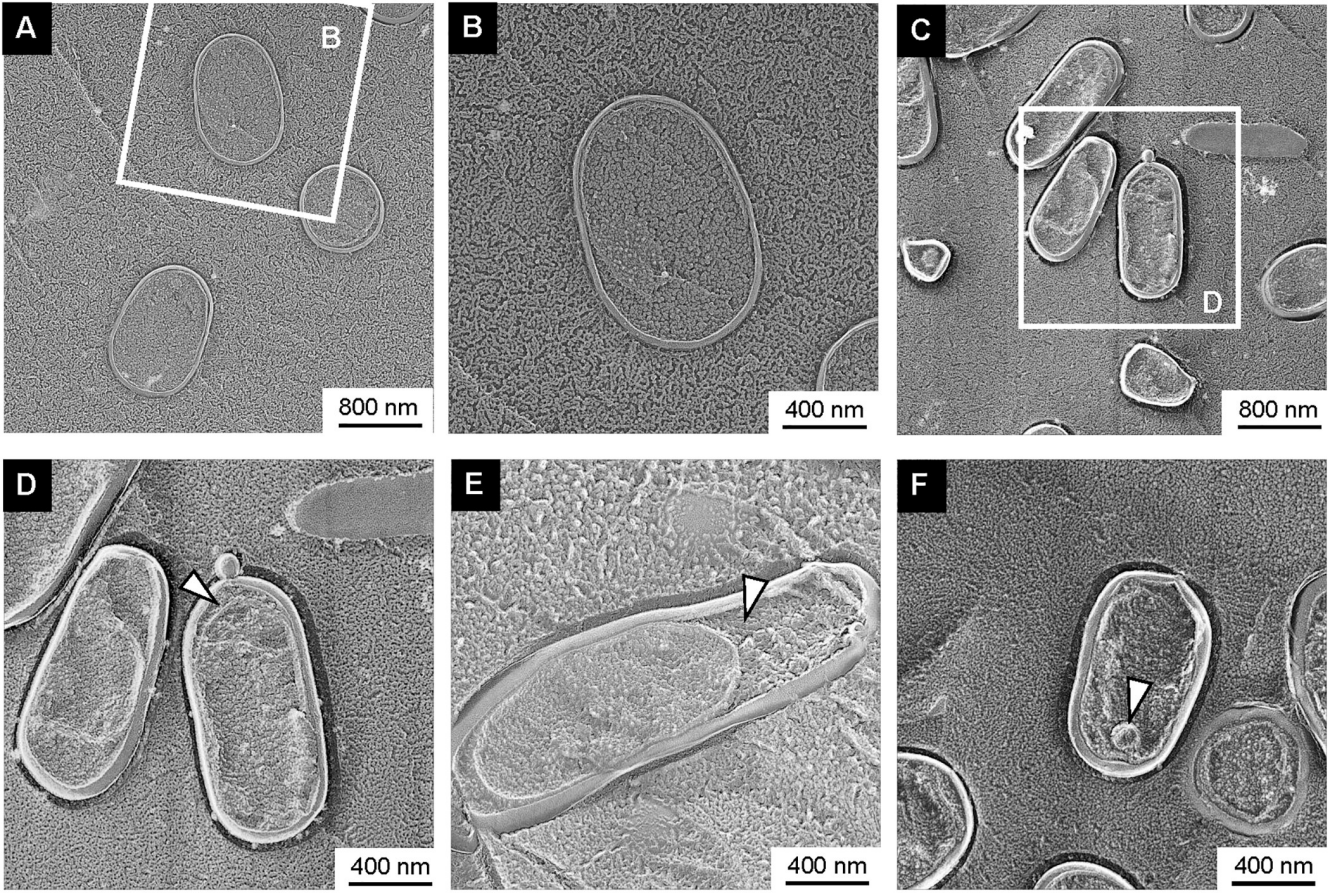
Cross-section of various fractured *E*. *coli* cells. (A) Field image of WT cells. (B) Magnified image of the structure of WT cell. (C) Field image of Δ*mlaE*Δ*nlpI* cells. (D–F) Magnified images of the structure of Δ*mlaE*Δ*nlpI* cells. The arrowheads indicate plasmolysis (D, E) or intracellular vesicle (F).

Freeze-fractured sections without the freeze-etching step visualized the inside of the cell structure (Fig 3). For example, the OM and inner membrane can be visualized, and the cytoplasmic and periplasmic spaces were inferred by this method. Compared with WT cells (Fig 3A and 3B), most EcN Δ*mlaE*Δ*nlpI* cells seemed to have large periplasmic spaces (Fig 3C). Magnified images revealed that the deletion of *mlaE* and *nlpI* induced frequent plasmolysis in the cells (Fig 3D and 3E). Plasmolysis is a process that occurs in plants and bacteria in which the protoplasm contracts away from the cell wall [21, 22]. Bubbling OMVs were found at the tip of the long axis of Δ*mlaE*Δ*nlpI* cells (Fig 3D), suggesting that the OMVs are mainly produced through plasmolysis. Intracellular vesicles were also observed in EcN Δ*mlaE*Δ*nlpI*, although in a small number (Fig 3F). The appearance of intracellular vesicles was reminiscent of PG damage in the Δ*mlaE*Δ*nlpI* of K12.

### Observation of sacculi using QFDE-EM and cell viability

**Fig 4:**
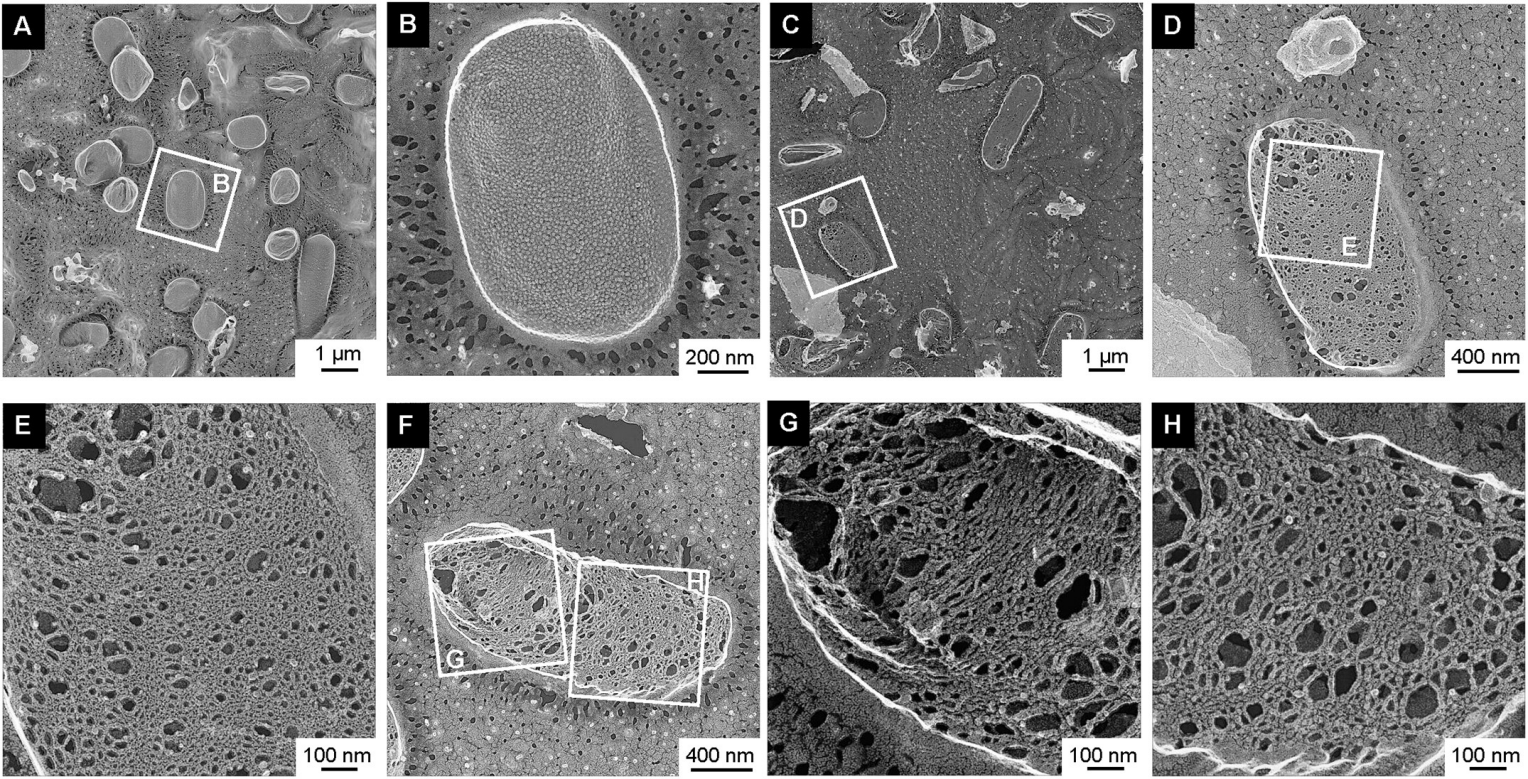
Sacculi of WT and Δ*mlaE*Δ*nlpI* EcN cells visualized by QFDE-EM. (A) Field image of the surface structures of WT sacculi. (B) A magnified image of WT sacculi. (C) Field image of the surface structures of Δ*mlaE*Δ*nlpI* sacculi. (D–H) Magnified images of Δ*mlaE*Δ*nlpI* sacculi.

We isolated sacculi from WT and Δ*mlaE*Δ*nlpI* of EcN to observe their surface structures using QFDE-EM. 10% SDS was used to dissolve the cell surface layer and exposed the peptidoglycan layer of the cell [15, 23]. The sacculi isolated from WT had a smooth and faultless surface (Fig 4A and 4B). The sacculi from the Δ*mlaE*Δ*nlpI* strain were longer on the long axis (Fig 4C). While in previous study the sacculi of the double gene knockout mutant of the *E*. *coli* K12 strain had holes at the tip of the long axis [15], the sacculi of EcN Δ*mlaE*Δ*nlpI* had countless holes throughout their surface (Fig 4D–4H). Magnified images showed that the Δ*mlaE*Δ*nlpI* sacculi had numerous holes and a porous structure like a sponge (Fig 4E, 4G and 4H). The diameter of the holes was up to ~100 nm. Because deep damage to sacculi causes cell death of bacteria [24], we evaluated the cell viability of EcN Δ*mlaE*Δ*nlpI*. Table 2 shows the number of colonies per OD_660_ after 24 h of culture.

**Table 2:** Colony forming units (cfu) per OD_660_ after 24 h culture.

Strain	Number of colonies per OD_660_ [10^7^ cfu/(mL•OD_660_)]	Ratio
WT	15.6	1
Δ*mlaE*Δ*nlpI*	5.5	0.36

The data were obtained from three independent experiments (*n* = 3).

The values for the WT and Δ*mlaE*Δ*nlpI* were 15.6 × 10^7^ and 5.5 × 10^7^ colony-forming units/mL•OD_660_, respectively. Assuming that all WT cells were live cells, even though ≥90% of the Δ*mlaE*Δ*nlpI* cells appeared to have numerous holes in the cells, more than one-third of the Δ*mlaE*Δ*nlpI* cells survived.

### Characterization of the sacculi from WT and Δ*mlaE*Δ*nlpI* cells

In Gram-negative bacteria, PG is composed of polysaccharide glycan chains and four amino acids [23]. To understand the degree of damage to PG in Δ*mlaE*Δ*nlpI* cells, the dry cell weight and the weight of dry sacculi were measured (Table 3).

**Table 3:** Dry weight of cells and sacculi in each EcN strain.

Strain	Dry cell weight (g/L)	Dry sacculus weight (g/L)	Weight percentage of dry sacculi in dry cells
WT	1.45 ± 0.14	0.14 ± 0.02	9.7
Δ*mlaE*Δ*nlpI*	1.07 ± 0.10	0.06 ± 0.01	5.7

The data were obtained from three independent experiments (*n* = 3).

The dry cell weight of the WT was 1.45 ± 0.14 g/L at 24 h and the dry sacculus weight was 0.14 ± 0.02 g/L; thus the weight percentage of sacculi in dry WT cells was 9.7%. In general, *E*. *coli* sacculi are known to be approximately 10% of the dry cell weight [25]. Meanwhile, because of the lower cell growth, the dry cell weight of the Δ*mlaE*Δ*nlpI* strain was 1.07 ± 0.10 g/L. The dry sacculus weight was 0.06 ± 0.01 g/L. Thus, the weight percentage of sacculi in Δ*mlaE*Δ*nlpI* cells was only 5.7%. These results suggest that the amount of sacculi decreased (in terms of weight) to around 60% of that in the WT. Considering the QFDE-EM images of sacculi in Fig 4, this value seems reasonable. We next examined the amino acid components of the PG by TLC and absorbance measurements with ninhydrin colorimetry. The same amount of sacculi (1 mg) prepared from each strain was hydrolyzed by HCl. In TLC, four bands appeared for D-alanine, L-alanine, D-glutamic acid, and 2, 6-diaminopimeric acid in both WT and Δ*mlaE*Δ*nlpI* (Date not shown). In addition to TLC results, absorbance measurements show that the total amounts of amino acids per total PG were almost same between WT and Δ*mlaE*Δ*nlpI* (Date not shown), suggesting that the amino acid components were not changed in Δ*mlaE*Δ*nlpI* compared to WT.

## Discussion

EcN, a probiotic strain of *E*. *coli*, is expected to be used in various medical applications such as vaccines, drug carriers, and regenerative medicine. Vesicles isolated from EcN play an important role in maintaining intestinal homeostasis, and they are called “postbiotics” having probiotic functions. In recent years, an increasing number of papers have been published on the antitumor effects of EcN and its application in vaccines. Although the cell surface layer of EcN differs from that of experimental *E*. *coli* strains in many respects, there are only a few papers focusing on the properties of the cell surface structure and its promotion mechanism of vesicle production.

In this study, a hypervesiculating EcN strain (Δ*mlaE*Δ*nlpI*) was constructed. The gene deletions of *mlaE* and *nlpI* enhanced OMV production in EcN. However, the increase was not as great as that previously observed for the equivalent mutations in *E*. *coli* K12 strain BW25113. The possible reason for the smaller increase in EcN compared with the K12 strain is the difference in cell surface structure. EcN has a different type of lipopolysaccharide (LPS) from *E*. *coli* K12. In the case of EcN, the O6 polysaccharide side-chain of LPS is very short compared to K12 strain and the *E*. *coli* O6 antigen repeat unit binds to the R1-type core, giving EcN a semi-rough phenotype. EcN also forms an extracellular K5 serotype capsular membrane [26]. These differences in cell surface structure may affect the OMV production. Further study is needed to elucidate detailed mechanisms of this effect.

Multilamellar vesicles were also observed in hypervesiculating EcN strain. Our previous studies have discussed the possibility of Δ*mlaE*Δ*nlpI* strains forming outer–inner membrane vesicles (OIMVs) [15]. There are holes in the PG of the Δ*mlaE*Δ*nlpI* because the deletion of the *nlpI* gene prevented the formation of typical numbers of Lpp-PG crosslinks. The cytoplasmic membrane material protrudes into the periplasmic space through the PG holes and is released as intracellular vesicles in Δ*mlaE*Δ*nlpI* cells at the first step. Next, the intracellular vesicles are released with the outer membrane, forming multilamellar vesicles. OIMVs are one of the multi-membrane vesicles composed of outer membranes and inner membranes, which contain DNA and other cytoplasmic components [27]. Multiple-membrane vesicles are considered to be one of the characteristics of hypervesiculating strains. For example, the *tolB* mutant of high vesicle-forming strain *Buttiauxella agrestis* JCM 1090T formed multiple-membrane vesicles [28]. Here we report that gene deletion of *mlaE* and *nlpI* resulted in formation of multiple-membrane vesicles not only in *E*. *coli* K12 strain but also in EcN.

In TEM observations of the EcN Δ*mlaE*Δ*nlpI* cell surface structure (Fig 2), the cells were elongate, but there was no significant damage to the cells or defects in the cell membrane. However, observation of cross-sectioned cells showed plasmolysis in the EcN Δ*mlaE*Δ*nlpI* cells and the extracted PG had numerous holes (Fig 4). While these phenotypes are similar to the findings for the Δ*mlaE*Δ*nlpI* of K12, there were more PG holes in the Δ*mlaE*Δ*nlpI* of EcN than the K12 strain, which were observed not only at the tip of the long axis but also in the whole PG structure. This difference seems related to the aforementioned difference in cell surface structure between EcN and K12 strains, but the detailed mechanism is still unclear and further study is required.

In general, PG damage is caused by incomplete PG synthesis or by PG-degrading enzymes. *In vitro*, the addition of glycine to culture medium above a certain concentration causes cell wall loss and cell destruction [24]. Hirayama and Nakao reported that the addition of 1.0% glycine induced hypervesiculation of EcN [12]. According to this report, the addition of 1.0% glycine caused a clear deformation of the cell structure and quasi-lysis, resulting in cell destruction and loss of PG 16 h after of the addition of glycine. However, in their study, the cell viability or the residual amount of PG in cells were not quantitatively determined. This study first revealed that Δ*mlaE*Δ*nlpI* cells of EcN had PG decreased to 60% of the amount in the WT, and one-third of the cells remained viable. SDS solution was also used to dissolve the cell surface layer and exposed the PG layer of the cell [15, 23]. It is possible that the PG originally had holes or that the 10% SDS treatment may have caused them, but the absence of defects in similarly treated WT sacculi suggested that Δ*mlaE*Δ*nlpI* sacculi were at least much weaker than WT sacculi. These results suggest that the residual amount of PG in Δ*mlaE*Δ*nlpI* of EcN is minimum level for growth in normal culture conditions.

Another type of *E*. *coli* cell that survives despite damage to PG is the wall-deficient L-formed cell [29]. When cell wall synthesis is inhibited in hypertonic medium with high Mg^2+^ under anaerobic conditions, the K12 cells swell from the center, pass through protoplast cells, and transform into amoeba-like L-formed cells. It was also reported that L-type *E*. *coli* cells retained only 7% of the total PG compared with normally grown cells [29]. Considering that the Δ*mlaE*Δ*nlpI* cells here maintained a rod-shape and had 60% of the PG compared with the WT, although their cell viability is considerably lower than that of WT, the Δ*mlaE*Δ*nlpI* cells are considered to be an intermediate type of cell between normal *E*. *coli* and L-type cells. Because the strain constructed here grew in normal culture conditions, its application as a probiotic is expected with OMVs serving as an immune booster.

## Conclusions

In this study, a hypervesiculating strain (Δ*mlaE*Δ*nlpI*) of the probiotic bacterium EcN was constructed and characterized. Similar to a comparable *E*. *coli* K12 strain, these EcN cells were elongate, and plasmolysis occurred inside the cells. Meanwhile there were more holes in the PG than the K12 strain, which were observed not only at the tip of the long axis but also in the whole PG structure. Although the amount of PG in Δ*mlaE*Δ*nlpI* cells decreased to approximately half of that in WT, the components of amino acids in PG did not change in Δ*mlaE*Δ*nlpI*. Although the viability decreased compared to the WT, the Δ*mlaE*Δ*nlpI* grew in normal culture conditions. The hypervesiculating EcN strain constructed here is expected to be applied as a probiotic strain using OMVs as an immune booster.

## Supporting information

S1 Table Primers used for knockout confirmation in this study. (DOCX)
