# Supplementary material for: Construction and characterization of a hypervesiculation strain of Escherichia coli Nissle 1917
Source: PLoS One. 2024 Apr 2;19(4):e0301613. doi: 10.1371/journal.pone.0301613 (PMC10986995; doi:10.1371/journal.pone.0301613)
Supplement: S1 Table — (DOCX) [file pone.0301613.s001.docx]

| Supplementary Table S1 Primers used for knockout confirmation in this study | |
| --- | --- |
| Primers | Sequences |
| *nlpI*-F | 5′- GGTAAAGGCGGTTCTGTAAT -3′ |
| *nlpI*-R | 5′- GCGTAATTGCACGTCATAAC -3′ |
| *mlaE*-F | 5′- CGCCTATCCACTGCGCGAAC -3′ |
| *mlaE*-R | 5′- CATTATTACCTGGCGCAGCA -3′ |
